# Fibrosis of extracellular matrix is related to the duration of the disease but is unrelated to the dynamics of collagen metabolism in dilated cardiomyopathy

**DOI:** 10.1007/s00011-016-0977-3

**Published:** 2016-08-11

**Authors:** Paweł Rubiś, Sylwia Wiśniowska-Śmialek, Ewa Wypasek, Barbara Biernacka-Fijalkowska, Lucyna Rudnicka-Sosin, Ewa Dziewiecka, Patrycja Faltyn, Lusine Khachatryan, Aleksandra Karabinowska, Artur Kozanecki, Lidia Tomkiewicz-Pająk, Piotr Podolec

**Affiliations:** 1 Department of Cardiac and Vascular Diseases, John Paul II Hospital, Pradnicka St. 80, 31-202 Krakow, Poland; 2 Department of Molecular Biology, John Paul II Hospital, Pradnicka St. 80, Krakow, Poland; 3 Department of Pathology, John Paul II Hospital, Pradnicka St. 80, 31-202 Krakow, Poland; 4 Jagiellonian University Medical College, Saint Ann St. 12, 31-008 Krakow, Poland

**Keywords:** Dilated cardiomyopathy, Extracellular matrix, Fibrosis, Markers, Collagen, Biopsy

## Abstract

**Background:**

Fibrosis of extracellular matrix (ECM) in dilated cardiomyopathy (DCM) corresponds to the myocardial over-production of various types of collagens. However, mechanism of this process is poorly understood.

**Objective:**

To investigate whether enhanced metabolism of ECM occur in DCM.

**Methods:**

Seventy consecutive DCM patients (pts) (48 ± 12.1 years, EF 24.4 ± 7.4 %) and 20 healthy volunteers were studied. Based on symptoms duration, pts were divided into new-onset (*n* = 35, 6 months) and chronic DCM (*n* = 35, >6 months). Markers of collagen type I and III synthesis-procollagen type I carboxy- and amino-terminal peptides (PICP and PINP) and procollagen type III carboxy- and amino-terminal peptides (PIIICP and PIIINP), collagen 1 (col-1), ECM metabolism controlling factors—tumor growth factor beta-1 (TGF1-β), connective tissue growth factor (CTGF), and ECM degradation enzymes—matrix metalloproteinases (MMP-2, MMP-9) and their tissue inhibitor (TIMP-1) were measured in serum. All pts underwent right ventricular endomyocardial biopsy to study ECM fibrosis.

**Results:**

The presence of fibrosis was detected in 24 (34.3 %) pts and was more prevalent in chronic DCM [17 (48.6 %) vs. 7 (20 %), *p* < 0.01]. The levels of PIIINP [4.41 (2.17–6.08) vs. 3.32 (1.69–5.02) ng/ml, *p* < 0.001], CTGF [3.82 (0.48–23.87) vs. 2.37 (0.51–25.32) ng/ml, *p* < 0.01], MMP-2 [6.06 (2.72–14.8) vs. 4.43 (2.27–7.4) ng/ml, *p* < 0.001], MMP-9 [1.98 (0.28–9.25) vs. 1.01 (0.29–3.59) ng/ml, *p* < 0.002)], and TIMP-1 [15.29 (1.8–36.17) vs. 2.61 (1.65–24.09) ng/ml, *p* < 0.004] were significantly higher in DCM, whereas levels of col-1 [57.7 (23.1–233.4) vs. 159.4 (31.2–512.9) pg/ml, *p* < 0.001] were significantly lower in DCM compared to controls. There were no differences in all measured serum markers of ECM metabolism between newonset and chronic DCM and as well as fibrosis positive and negative pts. Fibrosis was weakly correlated only with the duration of DCM (*r* = 0.23, *p* < 0.05), however, not a single serum marker of fibrosis correlated with fibrosis. Neither unadjusted nor adjusted models, constructed from serum markers of ECM metabolism, predicted the probability of myocardial fibrosis.

**Conclusions:**

Dynamics of ECM turnover in DCM is high, which is reflected by the increased levels CTGF and degradation enzymes. Synthesis of collagen type III prevailed over collagen type I. ECM metabolism was not different in DCM regardless of the duration of the disease and status of myocardial fibrosis. Serum markers of ECM metabolism were found not to be useful for the prediction of myocardial fibrosis in DCM.

## Introduction

The homeostasis of myocardial extracellular matrix (ECM) involves an ongoing cycle of synthesis and degradation of ECM components, mainly collagen, and is regulated by multiple factors, including mechanical stretch, neuro-hormonal system, and growth factors and cytokines, such as transforming growth factor beta (TGF-β) and connective tissue growth factor (CTGF) [1]. In the healthy left ventricle (LV) ECM replacement is about 5–9 %, however, in pathological conditions, e.g., after myocardial infarction, this figure can rise to 50 % [2]. ECM fibrosis is a hallmark of dilated cardiomyopathy (DCM) and is a consequence of collagen type I and III, which are major components of ECM, overproduction and accumulation in the interstitium and perivascular regions of the myocardium [3]. Synthesis of collagen type I and III requires cleavage by respective amino- and carboxy-terminal proteinases. As a result the carboxy- and amino-terminal pro-peptides of procollagen type I (PICP and PINP), and carboxy- and amino-terminal pro-peptides of procollagen type III (PIIICP and PIIINP) are released into the blood [4]. The appearance of those molecules was observed after myocardial infarction, hypertrophic cardiomyopathy, DCM or heart failure (HF) [5, 6].

Cardinal features of DCM, such as ventricular dilation and impairment of systolic function, are partially caused by the disintegration of collagenous network due to increased collagen metabolism, e.g., degradation by collagenolytic activity of matrix metalloproteinases (MMPs) and relative inefficiency of their tissue inhibitors (TIMPs) [7]. Elevated serum concentrations of collagen pro-peptides, especially PICP and PIIINP, have been associated with myocardial fibrosis and are considered markers of increased collagen turnover [8].

Although ECM fibrosis is imprinted in DCM pathology, still the process of fibrosis is not fully understood. Duration of DCM seems to be the crucial factor in the fibrosis development, however, paradoxically there are few studies that unequivocally showed such a relationship. Measuring blood markers of collagen metabolism indirectly provides insight into fibrosis at the myocardial level.

We hypothesized that the magnitude and dynamics of ECM fibrosis, assessed by means of blood markers, vary between new-onset and chronic DCM as well as between fibrosis positive and negative DCM.

## Methods

### Study group

From July 2014 to October 2015 we included 70 consecutive DCM patients who fulfilled pre-specified criteria and were willing to participate in the study. DCM was diagnosed according to the current European Society of Cardiology 2007 guidelines after an exclusion of significant coronary artery disease, primary heart valve disease, congenital heart disease, and arterial hypertension [9]. Based on detailed echocardiograms, all patients fulfilled strict morphological and functional criteria, e.g., all had dilated LV (>117 % of predicted LV end-diastolic diameter according to the Henry formula) and significantly depressed systolic function (LV ejection fraction <35 %) [10, 11]. All patients had stable HF symptoms, according to the New York Heart Association (NYHA) class I–III, for at least last 2 weeks. Based on the duration of symptoms, patients were divided into equal groups consisting of 35 subjects with new-onset (group 1, ≤6 months) and chronic (group 2, >6 months) DCM presentation. Duration of HF symptoms was defined as the time from the onset of subjective symptoms (dyspnea on exertion or at rest, paroxysmal nocturnal dyspnea, orthopnea, palpitations, and/or edemas) to the index hospitalization or ambulatory visit in cardiology clinics. Furthermore, the presence of concomitant non-cardiac diseases, such as bone and joint diseases, chronic liver insufficiency, peripheral atherosclerosis, and neoplasms, affecting collagen metabolism and the circulating levels of procollagens also served as exclusion criteria. The study protocol was approved by the relevant institutional committees and the ethical committee. All patients gave a written informed consent prior to the inclusion in the study.

### Echocardiography

All measurements were performed according to the recent recommendations of the European Associations of Cardiovascular Imaging [12]. Examinations were performed on commercially available equipment (Vivid 7 GE Medical System, Horten, Norway) with a phased-array of 1.5–4 MHz transducer and tissue Doppler imaging (TDI) software. The conventional M-mode, B-mode and Doppler parameters, including LV ejection fraction (EF) were calculated.

### Invasive studies

Coronary angiography was performed in all patients to exclude relevant coronary artery disease. Right heart catherization (RHC) was performed according to practices used at our center. Hemodynamic investigations were evaluated with balloon-floating catheter and Siemens Inc. Cathcor system. According to the Fick formula, cardiac output and index were calculated. Based on standard formulas, both systemic and pulmonary vascular resistance were calculated.

### Endomyocardial biopsy (EMB)

EMB procedures were performed by experienced operators via a femoral or jugular vein approach [13]. Long (104 cm), flexible, disposable biopsy forceps seven French size with small jaws (Cordis^®^, Johnson & Johnson Co, Miami Lakes, FL, USA) were used for the procedure. According to the F Fr size, forceps can remove tissue samples of ~1.85 mm per 2.46 mm^3^. Simultaneous fluoroscopic guidance and bioptom curvature enabled the precise biopsy of the right ventricular interventricular septum. Up to five myocardial samples were obtained, which were immediately stored in formalin (for light microscopic examinations) or snap-frozen in OCT-embedding medium and stored at −80 °C for immunohistochemical or molecular studies. The presence of fibrosis was determined qualitatively by an experienced pathologist blinded to the clinical data.

### Laboratory measurements

Venous blood samples were drawn on the day of the study after a 30-min supine rest in a fasting state in the morning. After centrifuge, supernatant was stored at −20 °C until assay. Concentration of collagen synthesis markers and markers of collagen degradation were determined in plasma using a commercially available ELISA tests as follows: collagen type 1 (manufactures’ reference values 46.7–178.9 pg/ml), procollagen I N-terminal propeptide (PINP 30.2–55.1 pg/ml), procollagen III N-terminal propeptide (PIIINP 2.69–63.56 ng/ml), procollagen I C-terminal propeptide (PICP 64–186 pg/ml = 0.064–0.186 ng/ml), procollagen III C-terminal propeptide (PIIICP 5.2–35.5 ng/ml), connective tissue growth factor (CTGF 2.3–42.5 ng/ml) (all from Cloud Clone Corp. Houston, TX, USA); RayBio MMP2 ELISA (~400 ng/ml), RayBio MMP9 ELISA (2.0–139.4 ng/ml), and RayBio TIMP-1 ELISA (up to 400 ng/ml) (all from RayBiotech, Norcross, GA, USA) and TGF-β (4.639–14.757 pg/ml = 4.639–14.757 ng/ml) (Diaclone SAS, Besancon Cedex, France). All measurements were performed by technicians blinded to the sample status. Intra-assay and inter-assay coefficients of variation were <7 %.

### Control group

We included 20 healthy individuals (age 35.8 ± 7.9 years, LV end-diastolic diameter 49.3 ± 3.4 mm, ejection fraction 63.8 ± 4.7 %) who volunteered to become the control group. The display of the control group serum markers of fibrosis results and manufactures’ reference values is displayed in the table below.

**Table:** 

Parameter	Healthy controls (*n* = 20)	Manufactures’ reference values
PICP (ng/ml)	0.23 (0.06–0.32)	0.064–0.186
PINP (pg/ml)	129.9 (75.6–448.1)	30.2–55.1
PIIICP (pg/ml)	187.2 (88.1–390.3)	5.2–35.5
PIIINP (ng/ml)	3.32 (1.69–5.02)	2.69–63.56
Col-1 (pg/ml)	159.4 (31.2–512.9)	46.7–178.9
TGF-β1 (ng/ml)	2.22 (0.97–4.74)	4.639–14.757
CTGF (ng/ml)	2.37 (0.51–25.32)	2.3–42.5
MMP-2 (ng/ml)	4.43 (2.27–7.4)	Up to 400
MMP-9 (ng/ml)	1.01 (0.29–3.59)	2.0–139.4
TIMP-1 (ng/ml)	2.61 (1.65–24.09)	Up to 400

### Statistical analysis

Distribution of variables was assessed with Shapiro–Wilk test. Comparisons of clinical parameters in groups were conducted with *t* Student test or with Mann–Whitney tests if lack of normality was found in. The same applies to comparison between fibrosis and no-fibrosis groups. Four groups of patients (new-onset DCM with or without fibrosis and chronic DCM with or without fibrosis) were compared using Kruskal–Wallis test, since again lack of normality was found in Shapiro–Wilk test. Impact of selected parameters on fibrosis probability was analyzed in logistic regression method. Three models were analyzed: unadjusted model, model with adjustment to disease duration and model with adjustment to disease duration, age, sex and BMI.

## Results

### Patients characteristics

Table 1 shows the baseline characteristics assessed in the two groups of patients with early (group 1, *n* = 35, ≤6 months) and chronic DCM (group 2, *n* = 35, >6 months). Early DCM patients had significantly smaller LV end-systolic diameter, measured in the parasternal long axis view, however, LV wall thickness, LV volumes, EF, and E/E’ were similar in both groups. The presence of fibrosis was detected in 24 (34.3 %) pts and was significantly more prevalent in chronic DCM. Pulmonary artery (PA) pressure and the presence of secondary pulmonary hypertension (PH) were comparable between two groups. Similarly, peak oxygen uptake (VO_2_peak) during cardiopulmonary exercise test was not different between groups. Patients with early DCM had higher concentration of hemoglobin, however, levels of CK-MB, troponin, hs-CRP, and NT-proBNP were similar. Both groups were on optimal HF pharmacotherapy. However, as expected patients with chronic DCM had significantly more ICDs and CRTs implanted.

**Table 1:** Baseline characteristics of the study population

Parameter	Group 1 (*n* = 35)	Group 2 (*n* = 35)	*p* value
Age (years)	48.5 ± 12	47.5 ± 12.3	0.73
Sex (male/female)	32 (91.4 %)/3 (8.6 %)	31 (88.6 %)/4 (11.4 %)	0.7
BMI (kg/m^2^)	27.7 ± 5	26.7 ± 5.7	0.4
NYHA class	2.49 ± 0.7	2.66 ± 0.76	0.33
Duration (months)	2.34 ± 1.4	46.3 ± 39.7	<0.001
LBBB (*n*, %)	10 (28.6 %)	8 (22.8 %)	0.3
LVESd/BSA (mm/m^2^)	28.3 ± 5.8	31.8 ± 7.9	0.04
LVEDd/BSA (mm/m^2^)	34.2 ± 5.3	37.1 ± 8.2	0.08
LVESvol/BSA (ml/m^2^)	91.9 ± 40.7	100.3 ± 56.2	0.48
LVEDvol/BSA (ml/m^2^)	122.7 ± 55.2	130.7 ± 64.5	0.59
EF (%)	24.5 ± 6.7	24.3 ± 8	0.91
E/E’ (average sep + lat)	21.3 ± 12.6	20.5 ± 10.5	0.78
ECM fibrosis (*n*, %)	7 (20 %)	17 (48.6 %)	0.01
PA mean (mmHg)	22.6 ± 10.1	23.6 ± 11.7	0.69
pH (*n*, %)	11 (33.3 %)	16 (45.7 %)	0.3
VO_2_peak (ml/kg/min)	18.3 ± 6.5	15.5 ± 5.7	0.14
Hb (g/dl)	14.54 ± 1.38	13.46 ± 1.74	0.006
CK-MB (U/l)	11 (8–28)	12 (8–29)	0.08
hs-troponin T (ng/ml)	0.0197 ± 0.0123	0.0251 ± 0.0223	0.2
hs-CRP (mg/dl)	4.22 (0.15–141)	1.89 (0.17–96)	0.18
NT-proBNP (pg/ml)	1505 (69–27,012)	2088.5 (73–33,530)	0.36
Beta-blocker (*n*, %)	34 (97 %)	35 (100 %)	0.3
ACE-I (*n*, %)	34 (97 %)	32 (91.5 %)	0.3
ARB (*n*, %)	1 (3 %)	1 (3 %)	1
MRA (*n*, %)	33 (94 %)	33 (94 %)	1
Furosemide (*n*, %)	18 (51.4 %)	24 (68.6 %)	0.14
CRT/ICD (*n*, %)	9 (25.7 %)	18 (51.4 %)	0.03

Data are presented as median (range), as mean ± SD or *n* (%)

### Comparison with controls

Comparison of serum ECM fibrosis parameters between DCM and control group is depicted in the Table 2. Among markers of collagen synthesis, only PIIINP was significantly higher in DCM, whereas PICP, PINP, and PIIICP were similar in DCM and controls. Furthermore, collagen-1 level was lower in DCM than in controls. CTGF levels were higher in DCM but level of TGF-β1 did not differ between DCM and controls. All indicators of increased ECM turnover, such as MMP-2, MMP-9 and TIMP-1 were significantly higher in DCM in comparison to control group.

**Table 2:** Serum values of ECM fibrosis parameters in DCM patients

Parameter	DCM (*n* = 70)	Control (*n* = 20)	*p* value
Age (years)	48.04 ± 12.1	35.8 ± 7.9	<0.001
LVEDd (mm)	71.4 ± 11.5	49.3 ± 3.4	<0.001
EF (%)	24.4 ± 7.4	63.8 ± 4.7	<0.001
PICP (ng/ml)	0.17 (0.06–0.46)	0.23 (0.06–0.32)	0.56
PINP (pg/ml)	129.9 (25.2–1050.2)	129.9 (75.6–448.1)	0.89
PIIICP (pg/ml)	211.8 (70.3–1165.1)	187.2 (88.1–390.3)	0.47
PIIINP (ng/ml)	4.41 (2.17–6.08)	3.32 (1.69–5.02)	0.001
Col-1 (pg/ml)	57.7 (23.1–233.4)	159.4 (31.2–512.9)	0.001
TGF-β1 (ng/ml)	2.26 (0.65–6.97)	2.22 (0.97–4.74)	0.81
CTGF (ng/ml)	3.82 (0.48–23.87)	2.37 (0.51–25.32)	0.01
MMP-2 (ng/ml)	6.06 (2.72–14.8)	4.43 (2.27–7.4)	0.001
MMP-9 (ng/ml)	1.98 (0.28–9.25)	1.01 (0.29–3.59)	0.002
TIMP-1 (ng/ml)	15.29 (1.8–36.17)	2.61 (1.65–24.09)	0.004

Data are presented as median (range)

### Comparison between early and chronic DCM

There were no differences in all measured serum markers of ECM metabolism between patients with new-onset and chronic DCM (Table 3). This means that synthesis of collagen type I (markers-PICP, PINP, collagen-1) was not increased in either early or chronic DCM, whereas synthesis of collagen type III (marker-PIIINP) was homogenously increased regardless of the duration of DCM. Moreover, increased ECM turnover (high serum values of MMP-2, MMP-9, and TIMP-1) was observed in both DCM groups. This also applies to the fibrosis controlling factor, such as CTGF, which was homogenously increased in both DCM cohorts. However, level of TGF-β1, which was similar in DCM and controls, was also evenly distributed in early and chronic DCM.

**Table 3:** Comparison of the serum ECM fibrosis parameters between early and chronic DCM

Parameter	Group 1 (*n* = 35)	Group 2 (*n* = 35)	*p* value
PICP (ng/ml)	0.18 (0.06–0.29)	0.15 (0.06–0.46)	0.52
PINP (pg/ml)	132.1 (31.2–625.2)	129.9 (25.2–1050.2)	0.43
PIIICP (ng/ml)	209.9 (77.2–1165.1)	213.7 (70.3–717.7)	0.79
PIIINP (ng/ml)	4.3 (2.4–5.37)	4.4 (2.17–6.08)	0.51
Col-1 (pg/ml)	67.44 (23.1–206.5)	55.96 (31.2–233.4)	0.42
TGF-β1 (pg/ml)	2.21 (1.21–6.31)	2.24 (0.65–6.9)	0.86
CTGF (ng/ml)	3.3 (0.48–12.4)	4.99 (0.65–23.9)	0.14
MMP-2 (ng/ml)	5.99 (2.7–12.89)	6.14 (2.97–14.83)	0.58
MMP-9 (ng/ml)	1.94 (0.28–9.25)	1.99 (0.29–6.96)	0.89
TIMP-1 (ng/ml)	15.79 (1.8–35.16)	14.88 (2.4–36.17)	0.74

Data are presented as median (range)

### Comparison between fibrosis positive and negative DCM

We compared serum fibrosis parameters in patients with and without ECM fibrosis (Table 4). It turned out that all measured fibrosis markers were at the similar level in both groups. Furthermore, based on the duration of DCM and presence of fibrosis, patients were divided into four groups: new-onset DCM without (*n* = 28) and with (*n* = 7) fibrosis and chronic DCM without (*n* = 18) and with (*n* = 17) fibrosis (Table 5). Similarly, no differences were observed in serum parameters of fibrosis between all four analyzed groups.

**Table 4:** Comparison of the serum ECM fibrosis parameters between ECM fibrosis positive and negative DCM patients

Parameter	ECM fibrosis negative (*n* = 46, 65.7 %)	ECM fibrosis positive (*n* = 24, 34.3 %)	*p* value
PICP (ng/ml)	0.18 (0.06–0.46)	0.16 (0.06–0.44)	0.88
PINP (pg/ml)	129.92 (25.2–625.2)	128.75 (31.2–1050.2)	0.7
PIIICP (ng/ml)	241.1 (83.51–165.1)	151.84 (70.3–717.7)	0.12
PIIINP (ng/ml)	4.41 (2.17–5.78)	4.21 (2.9–6.08)	0.94
Col-1 (pg/ml)	56.89 (23.1–206.5)	58.69 (27.9–233.4)	0.96
TGF-β1 (pg/ml)	2.18 (0.65–6.97)	2.24 (1.21–5.89)	0.32
CTGF (ng/ml)	3.39 (0.48–12.4)	4.3 (0.65–23.87)	0.44
MMP-2 (ng/ml)	6.06 (2.7–14.8)	6.06 (2.97–8.43)	0.99
MMP-9 (pg/ml)	2.03 (0.28–9.25)	1.59 (0.29–6.96)	0.45
TIMP-1 (pg/ml)	14.88 (1.8–35.16)	15.31 (2.1–36.17)	0.83

Data are presented as median (range)

**Table 5:** Comparison of serum markers of fibrosis between four groups of DCM patients, divided according to the duration of disease and fibrosis status

Parameter	Early DCM without fibrosis (*n* = 28)	Early DCM with fibrosis (*n* = 7)	Chronic DCM without fibrosis (*n* = 18)	Chronic DCM with fibrosis (*n* = 17)	*p* value
PICP (ng/ml)	0.19 (0.06–0.29)	0.15 (0.06–0.28)	0.15 (0.06–0.46)	0.17 (0.08–0.44)	0.89
PINP (pg/ml)	138.7 (44.7–625.2)	116.2 (31.2–221)	128.8 (25.2–594.9)	134.3 (48.1–1050.2)	0.56
PIIICP (ng/ml)	205.6 (83.5–1165)	209.9 (77.2–648)	258.5 (107.5–627.7)	147.5 (70.3–717.7)	0.49
PIIINP (ng/ml)	4.4 (2.4–5.4)	3.9 (3.1–5.01)	4.42 (2.17–5.78)	4.49 (2.92–6.08)	0.75
Col-1 (pg/ml)	72.3 (23.1–206.5)	57.7 (27.9–107.6)	47.8 (31.2–129.8)	59.7 (31.2–233.4)	0.48
TGF-β1 (ng/ml)	2.2 (1.36–6.31)	2.15 (1.2–5.88)	1.85 (0.65–6.97)	2.24 (1.49–5.73)	0.66
CTGF (ng/ml)	3.39 (0.48–12.4)	2.92 (1.2–11.98)	4 (1.44–11.75)	5.15 (0.65–23.8)	0.38
MMP-2 (ng/ml)	5.99 (2.7–12.9)	6.83 (4.56–7.24)	6.21 (3.5–14.8)	5.99 (2.97–8.43)	0.83
MMP-9 (ng/ml)	2.1 (0.28–9.24)	1.34 (0.34–5.9)	2.01 (0.58–5.77)	1.72 (0.29–6.9)	0.86
TIMP-1 (ng/ml)	16.3 (1.8–35.2)	15.3 (2.1–30.5)	14.39 (2.4–29.8)	15.9 (4.05–36.17)	0.96

Data are presented as median (range)

### No correlations between serum fibrosis markers and ECM fibrosis

Fibrosis was correlated only with the duration of DCM (*r* = 0.23, *p* < 0.05). However, not a single serum marker of fibrosis correlated with ECM fibrosis. Furthermore, fibrosis was not correlated with LV wall thickness, diameters, and volumes as well as EF, blood markers of myocardial necrosis (CK, CK-MB, troponin), marker of inflammation (hs-CRP) or myocardial strain (NT-proBNP).

### Probability of fibrosis

Finally, we verified whether there is an impact of serum markers of fibrosis on the probability of the ECM fibrosis (Table 6). Three models were constructed: unadjusted model (1), model with adjustment to the disease duration (2) and model with adjustments to the disease duration, age, sex and BMI (3). However, neither unadjusted nor adjusted models predicted the probability of ECM fibrosis.

**Table 6:** Models to predict the probability of ECM fibrosis

Parameter	Model 1				Model 2				Model 3			
	OR	95 % CI		*p*	OR	95 % CI		*p*	OR	95 % CI		*p*
PICP (ng/ml)	1.6	0.005	>100	0.87	0.99	0.003	>100	0.99	8.12	0.009	>100	0.54
PINP (pg/ml)	1.001	0.998	1.003	0.98	0.997	0.996	1.0031	0.88	0.999	0.996	1.003	0.72
PIIICP (ng/ml)	0.999	0.996	1.001	0.302	0.999	0.996	1.001	0.348	0.999	0.996	1.002	0.38
PIIINP (ng/ml)	1.001	0.999	1.005	0.9	0.998	0.999	1.004	0.76	0.998	0.999	1.0004	0.49
Col-1 (pg/ml)	1.002	0.99	1.01	0.65	1.001	0.99	1.01	0.85	0.99	0.98	1.01	0.72
TGF1-beta (pg/ml)	1.001	0.999	1.005	0.45	1.0024	0.9981	1.0068	0.27	1.0033	0.999	1.0081	0.17
CTGF (ng/ml)	1.072	0.937	1.227	0.309	1.057	0.922	1.211	0.429	1.067	0.929	1.226	0.35
MMP-2 (ng/ml)	0.925	0.731	1.171	0.517	0.878	0.684	1.128	0.309	0.861	0.663	1.119	0.26
MMP-9 (pg/ml)	0.9991	0.999	1.0002	0.525	0.9998	0.9995	1.0002	0.454	0.9999	0.999	1.0002	0.5
TIMP-1 (pg/ml)	1.0001	0.999	1.0006	0.67	1	0.9994	1.0005	0.922	0.9999	0.993	1.0005	0.7

Data are presented as median (range)

## Discussion

The main findings of the study are as follows: (1) blood marker of collagen type III synthesis was found to be increased in DCM whereas indices of collagen type I synthesis were not different compared to controls (2) all measured serum markers of ECM fibrosis did not differ between new-onset and chronic DCM as well as fibrosis positive and negative patients (3) none of the serum markers of fibrosis correlated or predicted the probability of ECM fibrosis.

### ECM fibrosis

The ECM undergoes fibrotic remodeling in the majority of various cardiac diseases and also as a function of age, resulting in a decreased myocardial compliance and altered functionality. Myocardial fibrosis present as two main patterns: reactive (or diffuse) fibrosis describes the expansion of collagen fibers without a significant loss of myocytes, and replacement (or reparative) fibrosis occurs when collagen is deposited in places of necrotic/apoptotic myocytes. Thus, reactive fibrosis is typically observed in DCM, hypertrophic cardiomyopathy or in the aging heart, whereas replacement fibrosis is commonly present after myocardial infarction, myocarditis or sarcoidosis [14]. Although in the broad terms ECM fibrosis can be divided into two aforementioned groups, nevertheless, specific patho-mechanisms leading to fibrosis greatly differ between various diseases [15]. Data on the fibrosis process in DCM come from few and small studies, usually from single, highly specialized centers. In a landmark study, Pauschinger et al. observed greater increase of collagen type I than type III deposition, with an overall increase in the collagen type I to III ratio in DCM [16]. Some subsequent studies confirmed the initial observation on the altered ratio of collagen type I and III in the myocardium. Increased myocardial deposition of collagen type I and III implies increased synthesis and high concentrations of metabolic by-products that are released into circulation. Measuring those blood molecules may help to understand the ECM remodeling.

### Collagen metabolism

As ECM fibrosis is an ongoing process in DCM, it is expected to observe persistently high blood levels of collagen pro-peptides. However, discordant findings on the collagen metabolism parameters were reported thorough the studies. So far, among numerous studied circulating molecules, only two collagen-derived serum peptides, such as PICP and PIIINP have been shown to be clearly associated with myocardial fibrosis [7, 17, 18]. Querejeta et al. found increased blood marker of collagen type I synthesis (PICP) in HF patients of hypertensive origin [17]. Similarly, Izawa et al. observed association between PICP and LV fibrosis in spironolactone treated DCM patients [18]. In line with this, Klappacher and colleagues showed association between PIIINP and ECM fibrosis in patients with ischemic and dilated cardiomyopathy [7]. On the other hand, Timonen et al. reported that serum levels of markers of collagen type I synthesis (PICP and PINP) were actually lower in patients than in controls, whereas markers of collagen type III synthesis (PIIICP and PIIINP) were higher in DCM patients [19]. Slightly different approach to study ECM metabolism was proposed by Sezen et al. who measured serum prolidase activity, an important exopeptidase involved in collagen turnover, and found lower blood level of prolidase in DCM than in healthy controls, which actually suggested decreased collagen turnover in DCM [20]. In our study, we observed that serum markers of collagen type I synthesis (PICP and PINP) did not differ between DCM and controls but PIIINP was significantly higher in DCM, which may suggest increased collagen type III synthesis. However, this issue seems to be more complex. Since, PICP is formed in a 1:1 stoichiometric ratio to the collagen type I molecule formed, the pro-peptide concentrations in blood are direct indicators of ongoing collagen type I synthesis [4, 5]. Therefore, observed lack of differences of PICP serum concentrations between DCM and controls clearly suggest low intensity of collagen type I synthesis. What is even more interesting, is comparable intensity of collagen type I synthesis in new-onset and chronic DCM, as well as in fibrosis positive and negative patients, as serum concentrations of PICP and PINP were not different between groups. The formation of collagen type III is slightly more complex as the cleavage at the amino-terminus proceeds at slower rate and, thus, partially processed procollagen molecules (p-N collagen type III molecules) can be found on the surface of collagen type III fibers. Although, blood concentration of PIIINP was found to be related to ECM fibrosis, it should be admitted that PIIINP concentrations in blood are not direct indicators of ongoing collagen type III synthesis [4, 5, 8]. Bearing this in mind, we found increased concentrations of PIIINP (but not PIIICP, which is less studied) in DCM in comparison to the controls but no differences between new-onset and chronic DCM and fibrosis positive and negative patients. Therefore, none of the studied serum markers of ECM metabolism helped to distinguish between new-onset and chronic DCM or fibrosis positive and negative DCM. Consequently, none of serum makers of collagen synthesis correlated or predicted the probability of fibrosis.

### ECM fibrosis controlling factors

The activation of renin-angiotensin-aldosterone system is a major determinant of fibroblast activation and collagen deposition, with TGFβ and CTGF acting as the sequential downstream signal mediators [1–3]. The role of TGFβ and CTGF in the pathogenesis of cardiac remodeling and HF has been extensively studied in the animal models [21]. However, performed studies in humans provided conflicting results. Increased expression of TGFβ and CTGF in hearts and in blood from patients with DCM, hypertrophic cardiomyopathy, and aortic stenosis has been confirmed but their relevance to ECM fibrosis remains unproven. In one study plasma TGFβ levels were found to be correlated with the LV myocardial expression of collagen type I mRNA in patients with severe aortic stenosis but no correlation was observed with invasively determined LV fibrosis [22]. Furthermore, it has been reported that plasma TGFβ was higher in controls than in patients with DCM [23]. This observation is in line with our results that plasma levels of TGFβ did not differ between DCM patients and the control subjects. Moreover, neither TGFβ nor CTGF differed between new-onset and chronic DCM or patients with and without fibrosis.

### Matrix metalloproteinases and tissue inhibitor (MMP/TIMP) system

In healthy conditions, the MMP/TIMP system remains in a dynamic balance, however, in pathological conditions, there is an over-expression of MMPs and down-regulation of TIMPs [24]. Numerous MMPs and TIMPs were found to be associated with long-term prognosis in HF and DCM but associations with cardiac fibrosis, although plausible from the pathologic point of view, have not been clearly demonstrated [25]. Sivakumar et al. found that myocardial content of MMP-2, MMP-9, TIMP-1, and TIMP-2 were significantly increased in DCM hearts compared to non-failing hearts [26]. Completely opposite data were provided by Batlle et al. who analyzed LV biopsies in advanced DCM and control heart donors and did not find any differences in the expression levels of MMP-1, MMP-2, MMP-3, and TIMP-1 between pathological hearts compared to control hearts [27]. Moreover, expression levels of MMP-9 were even down-regulated in diseased heart compared to controls. Only MMP-2 expression levels weakly correlated with fibrosis [27]. In line with this, Lopez and colleagues did not observe any correlations between serum levels of MMP-1, TIMP-1 or their ratio and invasively assessed fibrosis in HF patients [28]. In our study, we also did not find correlations between MMPs and TIMP-1 and cardiac fibrosis but we observed significantly higher values of MMP-2, MMP-9, and TIMP-1 in DCM than in controls, this suggests an ongoing ECM remodeling and increased collagen turnover in DCM. Interestingly, Picard et al. reported highest myocardial expression of MMP-1 and TIMP-1 in DCM patients with mildly dilated LV in comparison to those patients with either no LV dilatation or severe LV dilatation [29]. They concluded that the dynamics of LV dilatation and collagen turnover is highest in the early phases of cardiac remodeling. Although we did not measure myocardial expression of MMPs or TIMP, we found completely no differences in serum concentrations of MMP-2, MMP-9 and TIMP-1 in DCM patients regardless of duration of the disease or presence/absence of ECM fibrosis.

## Conclusions

We studied serum markers of fibrosis from various groups and points of action, including markers of collagen synthesis (PICP, PINP, PIIICP, PIIINP, collagen-1), fibrosis controlling factors (TGFβ and CTGF), and enzymes degrading collagen fibers (MMP-2, MMP-9, and TIMP-1) in homogenous DCM cohort with significant LV remodeling and LV systolic dysfunction. Based on our results, we speculate that once ECM remodeling in DCM starts (probably at early stages), its course and dynamics is homogenously increased in both new-onset or chronic DCM and as well as in fibrosis positive and negative DCM. Serum markers of collagen synthesis do not seem to be useful in the differentiation of new and chronic DCM or patients with or without fibrosis. Furthermore, serum markers of ECM metabolism were found not to useful for the prediction of myocardial fibrosis in DCM.

### Study limitations

This study has several limitations that should be acknowledged. The control group is significantly younger than the study group. However, the recruitment period of DCM patients lasted 12 months so the age- and sex-matched control group was found very difficult and impractical. Although we precisely defined the term—duration of symptoms (“Methods” section), that was used to differentiate patients into early and late DCM, we cannot completely rule out that some patients defined as new-onset DCM could have had milder HF symptoms that were unreported and not investigated. Because of the patchy distribution of myocardial fibrosis, the greatest potential limitation to endomyocardial biopsy evaluation is sampling error.
